# Supplementary figures and images for: Association between PM10 exposure and risk of myocardial infarction in adults: A systematic review and meta-analysis
Source: PLoS One. 2024 May 1;19(5):e0301374. doi: 10.1371/journal.pone.0301374 (PMC11062553; doi:10.1371/journal.pone.0301374)

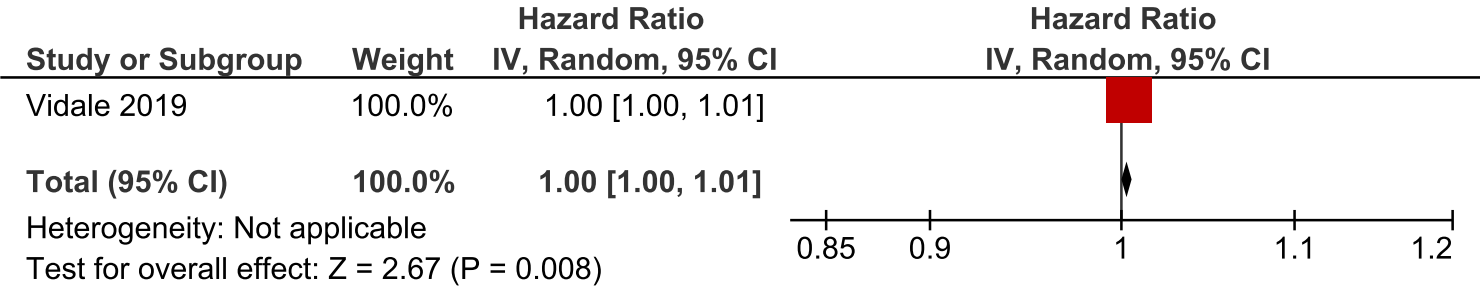

Supplement: S1 Fig — (PDF) [file pone.0301374.s001.pdf]

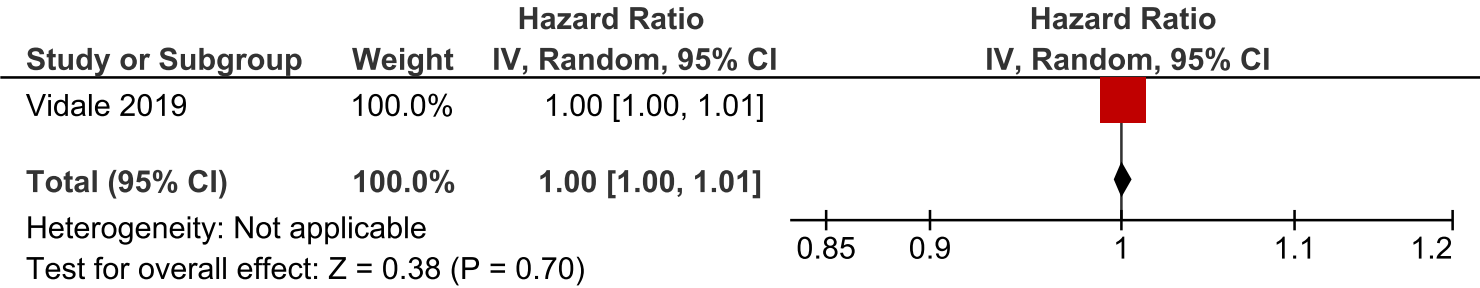

Supplement: S2 Fig — (PDF) [file pone.0301374.s002.pdf]
