# Supplementary material for: Association between PM10 exposure and risk of myocardial infarction in adults: A systematic review and meta-analysis
Source: PLoS One. 2024 May 1;19(5):e0301374. doi: 10.1371/journal.pone.0301374 (PMC11062553; doi:10.1371/journal.pone.0301374)
Supplement: S1 Table — (PDF) [file pone.0301374.s004.pdf]

#	Search Terms	OVID Medline	Embase	CINAHL	Web of Science
1	"Air pollution"	71,529	102,169	12,364	N/A
2	"Particulate matter"	39,216	72,632	5,438	N/A
3	"PM10"	8,899	14,177	1,009	N/A
4	1 or 2 or 3	92,580	147,058	14,492	214,878
5	"Myocardial infarction"	277,001	477,620	71,936	N/A
6	"Heart attack"	194,326	432,356	73,945	N/A
7	5 or 6	279,848	479,862	73,945	335,893
8	"Adult"	8,383,747	10,724,393	2,210,812	1,902,365
9	4 and 7 and 8	286	533	125	155
