# Supplementary material for: Association between PM10 exposure and risk of myocardial infarction in adults: A systematic review and meta-analysis
Source: PLoS One. 2024 May 1;19(5):e0301374. doi: 10.1371/journal.pone.0301374 (PMC11062553; doi:10.1371/journal.pone.0301374)
Supplement: S2 Table — (PDF) [file pone.0301374.s005.pdf]

#	Author/ publication year	Adjusted confounding variables
1	Argacha 2016	Age, comorbidities, smoking status, ambient air temperature, day of the week and season
2	Bard 2014	Holidays, meteorological variables (daily maximum temperature, maximum atmospheric pressure, and mean relative humidity), and influenza epidemics
3	Bhaskaran 2011	Daily mean temperature, daily relative humidity, daily levels of influenza and respiratory syncytial virus, day of week, and holidays
4	Buszman 2020	Unadjusted
5	Cheng 2021	Temperature, relative humidity, public holidays, circulating levels of influenza and respiratory syncytial virus
6	Claeys 2015	Unadjusted
7	Collart 2017	Seasonality, long-term trend, day of the week, and temperature
8	Davoodabadi 2019	Temperature, dew point, and wind speed
9	Downward 2018	Smoking status (including number of cigarettes and duration of smoking), diet (intake of fruit and vegetables), alcohol consumption, BMI, recruitment year, gender, marital status, education level, and area-level economic status
10	Huss 2010	Sex, civil status (single, married, divorced, widowed), nationality (Swiss, other), educational level (primary, secondary, tertiary), setting (urban, rural), language region (German, French, Italian), type of building (older than 30 years without renovation versus other), socioeconomic status of the municipality, noise, and distance.<br>Analysis restricted to persons who lived at least 15 years at the same place of residence.
11	Kim 2020	PM2.5, sex, age, income, smoking, alcohol, obese, physical activity, comorbidity for hypertension, diabetes, hyperlipidemia, area-level gross regional domestic products, % of high school graduated or more, % of elderly
12	Konduracka 2019	Temperature changes, relative humidity, and atmospheric pressure, infections prior to hospital admission
13	Kuzma 2021	Day of the week, public holidays, daily relative humidity, atmospheric pressure, and temperature
14	Lipsett 2011	Age, race, smoking status, total pack-years, body mass index, marital status, alcohol consumption, second-hand smoke exposure at home, dietary fat, dietary fiber, dietary calories, physical activity, menopausal status, hormone therapy use, family history of MI or stroke, blood pressure medication, and aspirin use, and for contextual variables (income, income inequality, education, population size, racial composition, and unemployment)
15	Nuvolone 2011	Age, gender, body mass index, influenza epidemics, population decreases during

		vacation periods, holidays, and apparent temperature
16	Puett 2008	Age in months, adjusting for state of residence, year, and season
17	Roye 2019	Influenza cases
18	Soleimani 2019	Hypertension, high blood pressure, relative humidity
19	Vidale 2017	Age, race, smoking status, total pack-years, body mass index, marital status, alcohol consumption, second-hand smoke exposure at home, dietary fat, dietary fiber, dietary calories, physical activity, menopausal status, hormone therapy use, family history of MI or stroke, blood pressure medication, and aspirin use, and for contextual variables (income, income inequality, education, population size, racial composition, and unemployment).
20	Yang 2022	Temperature, relative humidity, time, day of the week, and holiday
21	Yen 2022	Included gender, age group, low-income household, major injury card, Charlson comorbidity index, and meteorological data (daily temperature and relative humidity).
22	Zhang 2016	Temperature and relative humidity
23	Zhu 2019	Public holidays (Holiday) and day of week (DOW)
