# Supplementary material for: Association between PM10 exposure and risk of myocardial infarction in adults: A systematic review and meta-analysis
Source: PLoS One. 2024 May 1;19(5):e0301374. doi: 10.1371/journal.pone.0301374 (PMC11062553; doi:10.1371/journal.pone.0301374)
Supplement: S3 Table — (PDF) [file pone.0301374.s006.pdf]

#	Author/publication year	Study Design	Country	Sample size (n)	# Male (%)	Risk Measure	Exposure Increment	Lag Intervals
1	Akbarzadeh 2018 [37]	Case-crossover	Iran	208	182 (88.3)	RR	3.61μg/m^3	Lag 0: 24hrs post-exposure<br>Lag 1: Average of 3 weeks post-exposure
2	Atkinson 2013 [38]	Cohort	United Kingdom	13,956	8,471 (60.7)	HR	IQR change: 3μg/m^3	Lag 1: 5-year delay
3	Cheng 2009 [39]	Case-crossover	Taiwan	9,349	NR	OR	IQR change: 61.94μg/m^3	Lag 0-2: Average of same day and previous 2 days
4	Cramer 2020 [40]	Time-series	Denmark	22,882	0 (0)	HR	IQR change: 5.5μg/m^3	Lag 1: 1-year mean<br>Lag 2: 3-year mean
5	Kim 2017 [41]	Cohort	South Korea	136,094	66,851 (49.1)	HR	1μg/m^3	Lag 1: 7-year delay
6	Kuzma 2020 [42]	Case-crossover	Poland	1,790	1,172 (65.5)	OR	IQR change: 15.1μg/m^3	Lag 0: NR<br>Lag 1: NR
7	Lee 2017 [43]	Cohort	South Korea	37,880	26,787 (70.7)	HR	By Quintile: varying increments	Lag 0: 24hrs post-exposure
8	Liu 2020 [44]	Case-crossover	Canada	6,142	4,482 (73.0)	OR	IQR change: 16μg/m^3	Single-day lags: Lag 0, 1, 2<br>Multiple-day lags: Lag 0–3, 0–5
9	Pan 2019 [45]	Case-crossover	Taiwan	898	745 (83.0)	OR	IQR change: 50μg/m^3	Single-day lags: Lag 0, 1, 2, 3
10	Rasche 2018 [46]	Case-crossover	Germany	693	466 (67.2)	OR	10 ug/m^3	Single-day lags: Lag 1, 2, 3
11	Rodins 2020 [47]	Cohort	Germany	4,105	1,950 (47.5)	HR	1 ug/m^3	Lag 1: 14-year delay
12	Sahlen 2019 [48]	Case-crossover	Sweden	14,601	NR	OR	IQR change: 26.5μg/m^3	Lag 1-24: Hourly delays per lag

13	Sen 2016 [49]	Case-crossover	Turkey	402	310 (77.1)	RR	5 ug/m^3	Single-day lags: Lag 0, 1, 2, 7
14	Wang 2016 [50]	Case-crossover	China	972	515 (53.0)	OR	50 ug/m^3	Lag 0: Same day
15	Wichmann 2013 [51]	Case-crossover	Sweden	28,215	16,627 (58.9)	OR	IQR change: 10.4μg/m^3	Lag 0: Same day<br>Lag 1: 1-day delay<br>Lag 0-1: Average of same day and 1 day previous
16	Wichmann 2014 [52]	Case-crossover	Sweden	28,215	16,627 (58.9)	OR	IQR change: 10.4μg/m^3	Lag 0: Same day<br>Lag 1: 1-day delay<br>Lag 0-1: Average of same day and 1 day previous
17	Wolf 2015 [53]	Cohort	Germany	15,417	11,378 (73.8)	RR	24.3 ug/m^3	Lag 0: Same day<br>Lag 1: 1-day delay<br>Lag 0-5: Average of same day and previous 5 days
18	Yu 2018 [54]	Case-crossover	China	5,545	3,492 (63.0	RR	10 ug/m^3	Single-day lags: Lag 0, 1, 2, 3, 4, 5, 6<br>Multiple-day lags: Lag 0–1, 0–2, 0–3, 0–4, 0–5, 0–6
