# Supplementary material for: Association between PM10 exposure and risk of myocardial infarction in adults: A systematic review and meta-analysis
Source: PLoS One. 2024 May 1;19(5):e0301374. doi: 10.1371/journal.pone.0301374 (PMC11062553; doi:10.1371/journal.pone.0301374)
Supplement: S4 Table — (PDF) [file pone.0301374.s007.pdf]

ID#	Study	Q1	Q2	Q3	Q4	Q5	Q6	Q7	Q8	Q9	Q10	Q11	Q12	Q13	Q14	Score
**#226**	Akbarzadeh 2018	O	O	O	O	O	O	O	O	O	O	O	NA	O	O	Good
**#298**	Argacha 2016	O	O	O	O	O	O	O	O	O	O	O	NA	O	O	Good
**#379**	Atkinson 2013	O	O	O	O	O	O	O	O	O	O	O	NA	O	O	Good
**#355**	Bard 2014	O	O	O	O	O	O	O	O	O	O	O	NA	O	O	Good
**#415**	Bhaskaran 2011	O	O	O	O	O	O	O	O	O	O	O	NA	O	O	Good
**#129**	Buszman 2020	O	O	O	O	O	O	O	O	O	O	O	NA	O	–	Fair
**#89**	Cheng 2021	O	O	O	O	O	O	O	O	O	O	O	NA	O	O	Good
**#453**	Cheng 2009	O	O	O	O	O	O	O	O	O	O	O	NA	O	O	Good
**#44**	Claeys 2015	O	O	O	O	O	O	O	O	O	O	O	NA	O	–	Fair
**#288**	Collart 2017	O	O	O	O	O	O	O	O	O	O	O	NA	O	O	Good
**#140**	Cramer 2020	O	O	O	O	O	O	O	O	O	O	O	NA	O	O	Good
**#193**	Davoodabadi 2019	O	O	O	O	O	O	O	O	O	O	O	NA	O	O	Good
**#237**	Downward 2018	O	O	O	O	O	O	O	O	O	O	O	NA	O	O	Good
**#450**	Huss 2010	O	O	O	O	O	O	O	O	O	O	O	NA	O	O	Good
**#263**	Kim 2017	O	O	O	O	O	O	O	O	O	O	O	NA	O	O	Good
**#130**	Kim 2020	O	O	O	O	O	O	O	O	O	O	O	NA	O	O	Good
**#753**	Konduracka 2019	O	O	O	O	O	O	O	O	O	O	O	NA	O	O	Good
**#133**	Kuzma 2020	O	O	O	O	O	O	O	O	O	O	O	NA	O	–	Fair
**#105**	Kuzma 2021	O	O	O	O	O	O	O	O	O	O	O	NA	O	O	Good
**#265**	Lee 2017	O	O	O	O	O	O	O	O	O	O	O	NA	O	O	Good
**#424**	Lipsett 2011	O	O	O	O	O	O	O	O	O	O	O	NA	O	O	Good
**#64**	Liu 2020	O	O	O	O	O	O	O	O	O	O	O	NA	O	O	Good
**#428**	Nuvolone 2011	O	O	O	O	O	O	O	O	O	O	O	NA	O	O	Good
**#195**	Pan 2019	O	O	O	O	O	O	O	O	O	O	O	NA	O	O	Good
**#462**	Puett 2008	O	O	O	O	O	O	O	O	O	O	O	NA	O	O	Good
**#236**	Rasche 2018	O	O	O	O	O	O	O	O	O	O	O	NA	O	O	Good
**#137**	Rodins 2020	O	O	O	O	O	O	O	O	O	O	O	NA	O	O	Good
**#201**	Roye 2019	O	O	O	O	O	O	O	O	O	O	O	NA	O	O	Good
**#758**	Sahlen 2019	O	O	O	O	O	O	O	O	O	O	O	NA	O	O	Good
**#318**	Sen 2016	O	O	O	O	O	O	O	O	O	O	O	NA	O	O	Good
**#183**	Soleimani 2019	O	O	O	O	O	O	O	O	O	O	O	NA	O	O	Good

**#262**	Vidale 2017	O	O	O	O	O	O	O	O	O	O	O	NA	O	O	Good
**#312**	Wang 2016	O	O	O	O	O	O	O	O	O	O	O	NA	O	O	Good
**#380**	Wichmann 2013	O	O	O	O	O	O	O	O	O	O	O	NA	O	O	Good
**#367**	Wichmann 2014	O	O	O	O	O	O	O	O	O	O	O	NA	O	O	Good
**#347**	Wolf 2015	O	O	O	O	O	O	O	O	O	O	O	NA	O	O	Good
**#564**	Yang 2022	O	O	O	O	O	O	O	O	O	O	O	NA	O	O	Good
**#9**	Yen 2022	O	O	O	O	O	O	O	O	O	O	O	NA	O	O	Good
**#252**	Yu 2018	O	O	O	O	O	O	O	O	O	O	O	NA	O	O	Good
**#323**	Zhang 2016	O	O	O	O	O	O	O	O	O	O	O	NA	O	O	Good
**#759**	Zhu 2019	O	O	O	O	O	O	O	O	O	O	O	NA	O	O	Good
O = Criteria satisfied; – = Criteria unsatisfied; NA = Not applicable																
