# Supplementary material for: Association between PM10 exposure and risk of myocardial infarction in adults: A systematic review and meta-analysis
Source: PLoS One. 2024 May 1;19(5):e0301374. doi: 10.1371/journal.pone.0301374 (PMC11062553; doi:10.1371/journal.pone.0301374)
Supplement: S5 Table — O = Criteria Satisfied, -1 = Criteria Unsatisfied. (PDF) [file pone.0301374.s008.pdf]

**GRADE Criteria**	**RoB**	**Inconsistency**	**Indirectness**	**Imprecision**	**Publication Bias**	**Large magnitude of effect**	**Dose-response gradient**	**Residual Confounding**	**<u>Quality</u>**
**Result**	O	O	O	O	O	O	O	O	High
**Reason**	3 Good<br>0 Fair	I^2 = 60%	Already screened for	1.02 (0.97,1.07)<br>CI limits do not cross the 25th percentile.	<5 studies	NA	NA	NA	
