# Supplementary material for: Association between PM10 exposure and risk of myocardial infarction in adults: A systematic review and meta-analysis
Source: PLoS One. 2024 May 1;19(5):e0301374. doi: 10.1371/journal.pone.0301374 (PMC11062553; doi:10.1371/journal.pone.0301374)
Supplement: S6 Table — O = Criteria Satisfied, -1 = Criteria Unsatisfied. (PDF) [file pone.0301374.s009.pdf]

**GRADE Criteria**	**RoB**	**Inconsistency**	**Indirectness**	**Imprecision**	**Publication Bias**	**Large magnitude of effect**	**Dose-response gradient**	**Residual Confounding**	**<u>Quality</u>**
**Result**	O	–1	O	O	O	O	O	O	Moderate
**Reason**	4 Good<br>0 Fair	I^2 = 72%	Already screened for	1.01 (0.99, 1.03)<br><br>CI limits do not cross the 25th percentile.	<5 studies	NA	NA	NA	
