# Supplementary material for: Association between PM10 exposure and risk of myocardial infarction in adults: A systematic review and meta-analysis
Source: PLoS One. 2024 May 1;19(5):e0301374. doi: 10.1371/journal.pone.0301374 (PMC11062553; doi:10.1371/journal.pone.0301374)
Supplement: S10 Table — O = Criteria Satisfied, -1 = Criteria Unsatisfied. (PDF) [file pone.0301374.s013.pdf]

GRADE Criteria	RoB	Inconsistency	Indirectness	Imprecision	Publication Bias	Large magnitude of effect	Dose-response gradient	Residual Confounding	<u>Quality</u>
**Result**	O	O	O	O	O	O	O	O	High
**Reason**	5 Good<br>0 Fair	I^2 = 41%	Already screened for	1.01 (0.99, 1.02)<br><br>CI limits do not cross the 25th percentile.	5 studies<br><br>7 outcomes<br><br>Symmetrical	NA	NA	NA	
