# Supplementary material for: Association between PM10 exposure and risk of myocardial infarction in adults: A systematic review and meta-analysis
Source: PLoS One. 2024 May 1;19(5):e0301374. doi: 10.1371/journal.pone.0301374 (PMC11062553; doi:10.1371/journal.pone.0301374)
Supplement: S11 Table — O = Criteria Satisfied, -1 = Criteria Unsatisfied. (PDF) [file pone.0301374.s014.pdf]

**GRADE Criteria**	**RoB**	**Inconsistency**	**Indirectness**	**Imprecision**	**Publication Bias**	**Large magnitude of effect**	**Dose-response gradient**	**Residual Confounding**	**Quality**
**Result**	NA	NA	NA	NA	NA	NA	NA	NA	NA
**Reason**	1 Good<br>0 Fair	1 study	Already screened for	1.00 (1.00, 1.01)<br>CI limits do not cross the 25th percentile.	<5 studies	NA	NA	NA	
