# Supplementary material for: Association between PM10 exposure and risk of myocardial infarction in adults: A systematic review and meta-analysis
Source: PLoS One. 2024 May 1;19(5):e0301374. doi: 10.1371/journal.pone.0301374 (PMC11062553; doi:10.1371/journal.pone.0301374)
Supplement: S12 Table — O = Criteria Satisfied, -1 = Criteria Unsatisfied. (PDF) [file pone.0301374.s015.pdf]

GRADE Criteria	RoB	Inconsistency	Indirectness	Imprecision	Publication Bias	Large magnitude of effect	Dose-response gradient	Residual Confounding	Quality
**Result**	NA	NA	NA	NA	NA	NA	NA	NA	NA
**Reason**	1 Good<br>0 Fair	1 study	Already screened for	1.00 (1.00, 1.01)<br><br>CI limits do not cross the 25th percentile.	<5 studies	NA	NA	NA	
