# Supplementary material for: Association between PM10 exposure and risk of myocardial infarction in adults: A systematic review and meta-analysis
Source: PLoS One. 2024 May 1;19(5):e0301374. doi: 10.1371/journal.pone.0301374 (PMC11062553; doi:10.1371/journal.pone.0301374)
Supplement: S13 Table — O = Criteria Satisfied, -1 = Criteria Unsatisfied. (PDF) [file pone.0301374.s016.pdf]

**GRADE Criteria**	**RoB**	**Inconsistency**	**Indirectness**	**Imprecision**	**Publication Bias**	**Large magnitude of effect**	**Dose-response gradient**	**Residual Confounding**	**<u>Quality</u>**
**Result**	O	–1	O	O	O	O	O	O	High
**Reason**	6 Good<br>0 Fair	I^2 = 6%	Already screened for	1.00 (0.99, 1.01)<br><br>CI limits do not cross the 25th percentile.	6 studies<br><br>6 outcomes<br><br>Symmetrical	NA	NA	NA	
